# miRNA Transcriptome of Hypertrophic Skeletal Muscle with Overexpressed Myostatin Propeptide

**DOI:** 10.1155/2014/328935

**Published:** 2014-07-24

**Authors:** Ruheena Javed, Lu Jing, Jinzeng Yang, Xinyun Li, Jianhua Cao, Shuhong Zhao

**Affiliations:** ^1^Department of Animal Genetics & Breeding, College of Animal Science and Veterinary Medicine, Huazhong Agricultural University, Wuhan, Hubei Province 430070, China; ^2^Department of Human Nutrition, Food and Animal Sciences, University of Hawaii at Manoa, Honolulu, HI 96822, USA

## Abstract

MicroRNAs (miRNAs) play an imperative role in cell proliferation, differentiation, and cell metabolism through regulation of gene expression. Skeletal muscle hypertrophy that results from myostatin depression by its propeptide provides an interesting model to understand how miRNA transcriptome is involved in myostatin-based fiber hypertrophy. This study employed Solexa deep sequencing followed by Q-PCR methods to analyze miRNA transcriptome of skeletal muscle of myostatin propeptide transgenic mice in comparison with their littermate controls. A total of 461 mature known and 69 novel miRNAs were reported from this study. Fifty-seven miRNAs were expressed differentially between transgenic and littermate controls, of which most abundant miRNAs, miR-133a and 378a, were significantly differentially expressed. Expression profiling was validated on 8 known and 2 novel miRNAs. The miRNA targets prediction and pathway analysis showed that FST, SMAD3, TGFBR1, and AcvR1a genes play a vital role in skeletal muscle hypertrophy in the myostatin propeptide transgenic mice. It is predicted that miR-101 targeted to TGFBR1 and SMAD3, miR-425 to TGFBR2 and FST, and miR-199a to AcvR2a and TGF-*β* genes. In conclusion, the study offers initial miRNA profiling and methodology of miRNA targets prediction for myostatin-based hypertrophy. These differentially expressed miRNAs are proposed as candidate miRNAs for skeletal muscle hypertrophy.

## 1. Introduction

Skeletal muscle growth and maintenance are essential for animal and human health as 40–60% of the total body mass is composed of skeletal muscles, which provide structural support and enable the body to maintain posture, to control motor movements, and to store energy [1]. Skeletal muscle displays a remarkable plasticity with great capacity to alter its size, structure, and function in response to various stimuli. It therefore plays a vital role in the whole body metabolism. The muscle fibers are established early in life, and muscle growth and development are regulated by an interesting protein named myostatin, a cytokine synthesized in skeletal muscle cells. Myostatin (MSTN), also known as growth and differentiation factor-8 (GDF-8), is a member of the transforming growth factor-*β* (TGF-*β*) superfamily and was identified by McPherron et al. [2]. Myostatin is expressed in skeletal muscle tissue where it functions to suppress myoblast proliferation and myofiber hypertrophy [3]. Genetic disruption of myostatin causes a dramatic increase in muscle mass. MSTN binds to activin receptor type IIB (ActRIIB) to exert its biological activity [4–6]. The binding of MSTN to its receptor leads to phosphorylation of transcription factors Smad2 and Smad3 along with subsequently forming a complex with Smad4, resulting in nuclear translocation of the Smad complex and consequent regulation of transcription of downstream target genes [7–9].

Myostatin mRNA is conserved across many mammalian species and massive muscle growth is observed in several livestock animal species with mutations in* MSTN* gene [10]. Previously, studies reported that MSTN-knockout mice show muscle fiber hyperplasia and hypertrophy in comparison with wild type mice [2]. Like other TGF-*β* family members, myostatin is synthesized as a precursor protein, which undergoes two posttranslational cleavage events, generating an N-terminal and a C-terminal peptides. The N-terminal peptide is referred to as myostatin propeptide while the C-terminal peptide is the actual mature form of myostatin. Transgenic overexpression of myostatin propeptide cDNA in skeletal muscle increases animal growth and muscle mass [11, 12]. Enhanced muscle mass phenotype in the propeptide transgenic mice primarily results from myofiber hypertrophy rather than myofiber hyperplasia. The size of fast-twitch, glycolytic muscle fiber at 9 weeks of age was increased by 60% compared with wild-type littermates [11]. By systemic comparisons of global mRNA expression in hypertrophic muscle of the transgenic mice using microarray and qRT-PCR techniques, the distinct expression patterns are identified which are comprised of enhanced expressions of myogenic regulatory factors and extracellular matrix components and differentially downregulated expressions of genes related to protein degradation and mitochondrial ATP synthesis [13, 14].

MicroRNAs are reported to act as negative regulators of target gene expression by recruiting silencing complexes to complementary sequence elements in target mRNAs. miRNAs are small 19–24 nucleotide (nt) RNAs that generally modulate gene expression through translational repression or by causing deadenylation and degradation of target mRNAs [15, 16]. Bioinformatic studies have demonstrated that each miRNA has hundreds of target genes in animals, and up to 30% of all animal genes are miRNA targets [17–22]. Previous studies demonstrated that miRNAs play an essential role in skeletal muscle development by regulating gene expression. In skeletal muscle, miRNAs have been implicated in proliferation, differentiation, hypertrophy, regeneration, and disease [23]. They are functionally significant and potentially key regulators of gene expression during skeletal muscle development [24–29]. For example, deletion of a conditional Dicer allele in embryonic skeletal muscle results in perinatal lethality due to skeletal muscle hypoplasia [30]. The pivotal roles of three muscle-specific miRNAs, miR-1, miR-133, and miR-206, in the regulation of myogenesis have been well documented [17, 31, 32]. A group of miRNAs, highly enriched in skeletal muscle (referred to as myomiRs), has recently been identified and includes miR-1, miR-133a, miR-133b, miR-206, miR-208, miR-208b, miR-486, and miR-499 [33–37]. Several of these miRNAs are organized under bicistronic clusters on the same chromosome (i.e., miR-1-1/133a-2, miR-1-2/133a-1, and miR-206/133b) and are transcribed together [17, 38, 39]. Regulation of these myomiRs is controlled by key myogenic regulatory factors (MRFs), including myogenic differentiation 1 (MyoD) and myogenin [40, 41] as well as myocyte enhancer factor 2 (MEF2) [42], serum response factor (SRF) [23], and myocardin-related transcription factor-A (MRTF-A) [33]. MyomiRs influence multiple facets of muscle development and function through regulation of the key myogenic genes [23, 40, 43]. Recent studies demonstrated that miR-29b, miR-133a, and 133b regulate myoblast proliferation and differentiation [38, 44], and miR-1 and miR-133 have been reported to regulate different aspects of skeletal muscle development* in vitro* and* in vivo* [23]. miR-1 promotes myocyte differentiation by repressing the expression of histone deacetylase 4 (HDAC4), a negative regulator of differentiation and a repressor of the MEF2 (myocyte enhancer factor-2) transcription factor [23].

*MSTN* gene also can regulate certain miRNAs expression through feedback autoregulation mechanism [45]. In Piedmontese cattle with mutated myostatin gene,* MSTN* gene was found to be targeted by miR-27b and miR-27b expression was correlated with hypertrophic phenotype of Piedmontese cattle [46]. A recent study further confirmed that MSTN regulates miR-27a/b via SMAD3 in feedback autoregulation manner. Increased expression of miR-27a/b was correlated with decreased expression of MSTN and vice versa both* in vitro* and* in vivo* mouse model. Loss of SMAD3 was associated with an increased level of MSTN [47].

Although the functional significances of miRNAs in controlling myogenesis have been documented and the majority of miRNAs are abundantly expressed, identifications of novel miRNAs that are expressed at low levels during skeletal muscle development have not been well defined. With robust approaches such as high-throughput deep sequencing technology, we can produce the whole miRNA transcriptomes [48]. In this study, we have attempted to identify candidate miRNAs in hypertrophic skeletal muscle with transgenic overexpressed myostatin propeptide by using transcriptome deep sequencing. Sequencing results have shown that a group of highly abundant known miRNAs were expressed in skeletal muscle. Using computational target prediction software we have identified differentially expressed miRNAs associated with muscle hypertrophy. An interaction network between miRNA and identified targets was constructed.

## 2. Materials and Method

### 2.1. Muscle Tissue Sample Collection

Myostatin propeptide transgenic mice were generated by standard microinjection techniques using B6SJL F1 females (Taconic, Germantown, NY) as zygote donors using the transgene MLC-pro construct [11]. The MLC-pro construct consists of myosin light chain (MLC) promoter and enhancer, myostatin cDNA from the 50 untranslated regions to the nucleotides encoding the propeptide cleavage site (RSRR), and SV40 PolyA tail signal sequence. Transgenic mice and wild-type littermate mice were obtained from offspring of MLC-pro transgenic mice mating with B6SJL F1. All procedures and animal care were in accordance with the institution guidelines and approved by the Institutional Animal Care and Use Committee of the University of Hawaii, Hawaii, USA. Myostatin propeptide-transgenic mice were generated by standard microinjection techniques, which has been previously described [9]. Male mice (hemizygous genotype for the transgene) from the high-expressing line were mated with B6SJL F1 wild-type females to produce offspring mice, which were used in this study. Mice were housed in cages; room temperature was maintained at 22°C and 12 h light/dark cycle. Mice were weaned at 4 weeks of age and given free access to a chow diet (10% kcal fat, ME3.85 kcal/g). Male mice at 4 months age were sacrificed for muscle tissue dissections and sampling after 8 hr fasting. Gastrocnemius muscle samples from both legs were immediately dissected from carcass, cleaned from fat, blood and quickly frozen in liquid nitrogen, and later stored at −80°C. Two wild-type littermate control samples, that is, CN148 and CN150, and three transgenic mouse samples, that is, TN126, TN135, and TN 329, were, respectively, collected from gastrocnemius muscle. Samples were immediately frozen in liquid nitrogen and stored at −80°C freezer.

### 2.2. Small RNA Library Preparation

Total RNA was isolated using TRIzol reagent (Invitrogen). Approximately 5 *μ*g of total RNA from transgenic and control mice was submitted to the Beijing Genomics Institute (BGI) for Solexa sequencing. In brief, polyacrylamide gel electrophoresis (PAGE) technique was used to fractionate total RNA in the range of 16–30 nt, for sequencing. Fragments were then ligated with proprietary adapters. cDNA was synthesized from total RNA by reverse transcription and amplified to produce libraries for sequencing.

### 2.3. Analysis of the Output Data

The data as obtained in fastq format was converted to fastq2fast.pl; and revalidated clean miRNA-seq dataset was analyzed using mirDeep software v.2.1.2. The* Mus musculus* genome* Mus_musculus. *GRCm38.74 was downloaded from Ensembl, and the miRNA reference was obtained from the miRBase database (version 20).

The miRNA expression level of each library was normalized by the following formula:

(1) Normalized  reads  count =Reads  count×1,000,000  total  clean  reads  count.

The Student's *t*-test was used to calculate the *P* value and analyze the expression difference between different groups [59]. The *q* value was calculated by fdrtool in *R* package [60].

### 2.4. Novel miRNA Prediction

The known and novel miRNAs were identified from deep sequencing data using miRDeep software v.2.1.2 package [61]. All sequences were mapped to the mouse genome using megaBLAST, and exactly matched sequences were processed for further analysis. Potential precursor miRNA sequences were extracted from the mouse genome and the secondary structures were predicted by RNAfold [62] (Figure 4).

### 2.5. Target Prediction

To observe the potential function of miRNAs with significantly differential expression in two groups (transgenic versus wild-type control), we used miRDB (Version 6.2) to predict putative target genes of miRNAs with homologous human miRNAs and target prediction score ≥ 60 [63] (http://mirdb.org/miRDB/index.html). Validated targets module of miRWalk was also used to investigate hosts experimentally verified target genes of differentially expressing miRNAs in the study (http://www.umm.uni-heidelberg.de/apps/zmf/mirwalk/) [64].

### 2.6. Gene Ontology and Pathway Analysis

Gene ontology analysis was performed by analyzing KEGG pathways using “database for annotation, visualization, and integrated discovery” (DAVID) website [65, 66] with the following parameters: Count = 2 and EASE = 1.0. “Count” means the threshold of minimum gene counts belonging to an annotation term, and “EASE” is a modified Fisher Exact *P* value.

## 3. Results

### 3.1. Overview of the Sequencing Data

**Figure 1:**
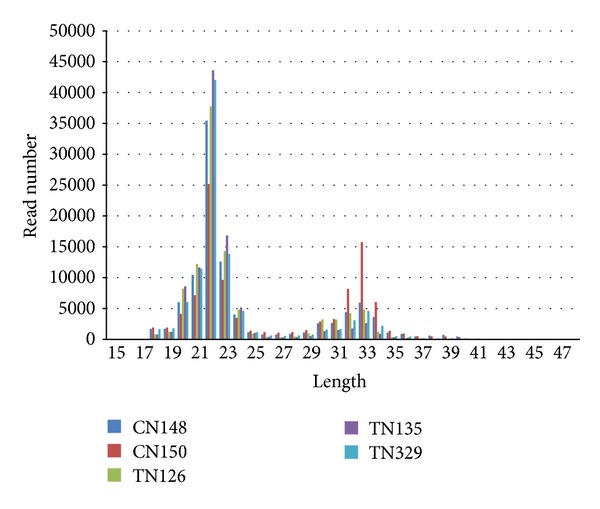
miRNA length distribution graph: miRNA length distribution graph depicting that most of all miRNA was consistent length of 22 nt.

**Figure 2:**
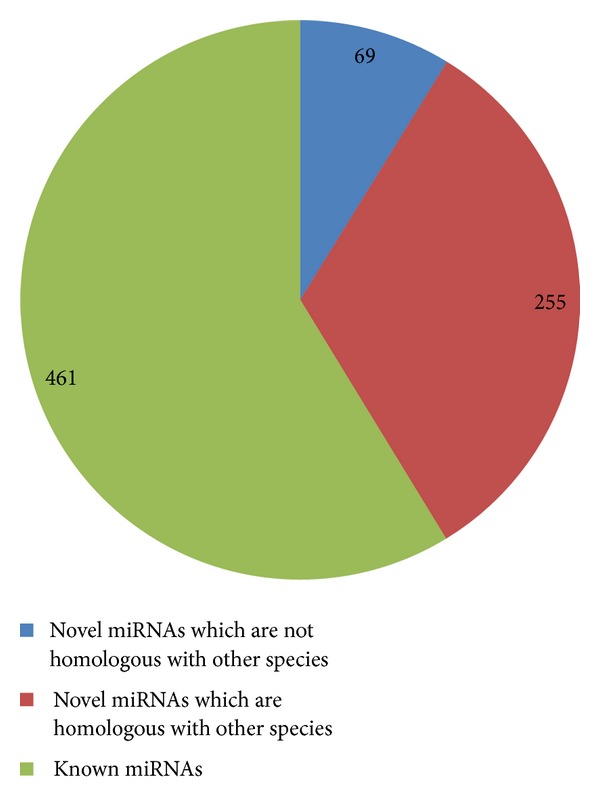
Annotation of miRNAs showing the number of known, homologous novel, and nonhomologous miRNAs.

Total reads from control CN148-11303077 and CN150-9357529 and from MSTN transgenic mice TN126-9734519, TN135-10207035, and TN329-21774919 were obtained. After trimming the adaptor and low quality reads, only clean reads of high quality were used for further analysis, that is, 10479962-CN148, 8375356-CN150, 10130133-TN126, 9052728-TN135, and 9677365-TN329 accounting for 92.72%, 89.50%, 93.24%, 92.99%, and 94.81%, respectively, of the original reads. The sequencing data were simplified by grouping all identical sequence reads together; therefore 536489 (CN148), 224550 (CN150), 228238 (TN126), 177471 (TN135), and 190093 (TN329) unique sequences were used for subsequent analysis (Table 1). The most abundant size class in small RNA sequences distribution was 22 nt, followed by 21 and 23 nt (Figure 1), and these estimates were in the accordance with the known 21–23 nt range for miRNAs [67]. To assess the efficiency of deep sequencing for miRNA detections, all sequence reads were annotated and classified by analyzing the sequence tags in relation to the data from miRBase (miRBase V20). The sequence tag annotation demonstrated that known* Mus muculus* miRNAs accounted for 59% of all sequence reads in the MSTN and control libraries (Figure 2). These results indicate that the deep sequencing data were highly enriched for mature miRNA sequences, suggesting that the data are reliable for expression profiling of known miRNAs and deep mining for novel miRNAs.

Comparative expression of miRNAs in transgenic and control mice skeletal muscles revealed that 461 miRNAs obtained in the sequencing data matched perfectly with 1908 known* Mus musculus* miRNAs in the miRBase.

### 3.2. miRNA Transcriptome Analysis Demonstrated the Presences of Several Highly Abundant miRNAs in Mouse Skeletal Muscle

Almost all known muscle-specific miRNAs (myomiRs) of the more abundant miRNAs were identified in the analyzed muscle samples. The most abundant expressed miRNA was mmu-miR-22a which was represented by approximately 1637847 and 924589 reads in the small RNA libraries of the wild-type mice (CN148 and CN150) and 755381, 1123582, and 3994285 reads in the small RNA libraries of transgenic mice (TN126, TN135, and TN 329), respectively (Table 2, see Additional File 1 in the Supplementary Materials available online at http://dx.doi.org/10.1155/2014/328935). The predominance of miR-133a was consistent with its well established function during skeletal muscle development; miR-378a is also reported to play important role during* Mus musculus* myogenesis. Two other myomiRs, miR-26a, and miR-27b were high-count sequences in both libraries of transgenic and wild-type mice. MyomiR 208 and 499 were expressed at a very low level in current study.

### 3.3. Identification of miRNA Isoforms

**Figure 3:**
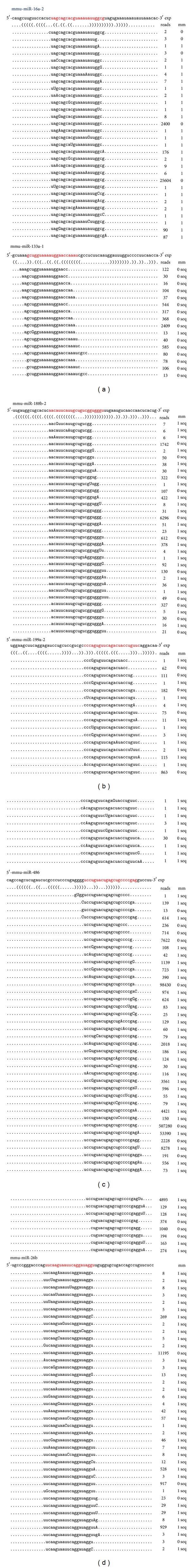
IsomiRs from different miRNAs: reads alignments of the various isoforms of several mmu-miRs are presented. The sequence of the mmu-miR hairpin is presented in the top line; the brackets below denote the secondary structure. Reads aligned with the mature mmu-miR sequence as reported in miRBase are denoted by a series of asterisks. The number of reads corresponding to each sequence is presented on the right.

The miRNAs also represent heterogeneity at 5′ and/3′ end (Figure 3) and these variations from their miRBase reference sequences are referred to as isomiRs [68, 69]. Examples of isomiRs are presented in Figure 3. In one case, the majority isoform, mmu-miR-16a-2, the most abundant isoform is identical to the reference in miRBase (Figure 3, Additional File 2). In some other cases, such as mmu-miR-181b-2, mmu-miR-199a-2, and mmu-miR-16a-2, more than one highly abundant isoform was present (Figure 3), indicating that some miRNAs have more than one isoformin specific tissues.

### 3.4. Novel miRNAs Were Less Abundant and Less Conserved

**Figure 4:**
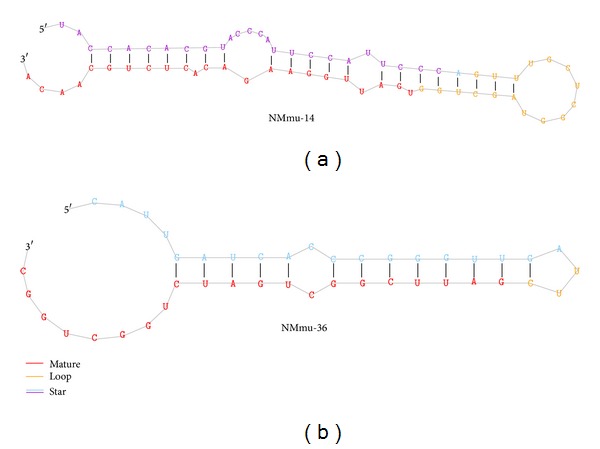
Predicted secondary structure of novel miRNAs: novel miRNAs secondary structure was predicted using miRDeep Software, where red color indicates the mature sequence, yellow color indicates loop sequence, blue color indicates the predicted star sequence, and purple color indicates miRNA star sequence.

In addition to profiling known miRNAs, deep sequencing is a powerful strategy for discovering novel miRNAs that may not have been detected by traditionalmethods from sequencing cDNA libraries. We identified a total of 69 putative novel* Mus musculus* miRNAs from wild-type controls and transgenic mouse sequence tags (Additional File 3). The putative novel miRNAs were less abundant than known miRNAs (Additional File 3). Two novel miRNAs NMmu-14 and NMmu-36 having more than 100 read counts were validated and NMmu14 was found to be relatively abundant in comparison with miR NMmu36 (Figure 4) but both of these had read counts greater than 100 in the small RNA library from TN 329 library (Table 3). The novel miRNAs were less evolutionarily conserved (Additional File 3).

### 3.5. Identifications of Differentially Expressed miRNAs in Hypertrophic Muscle

**Figure 5:**
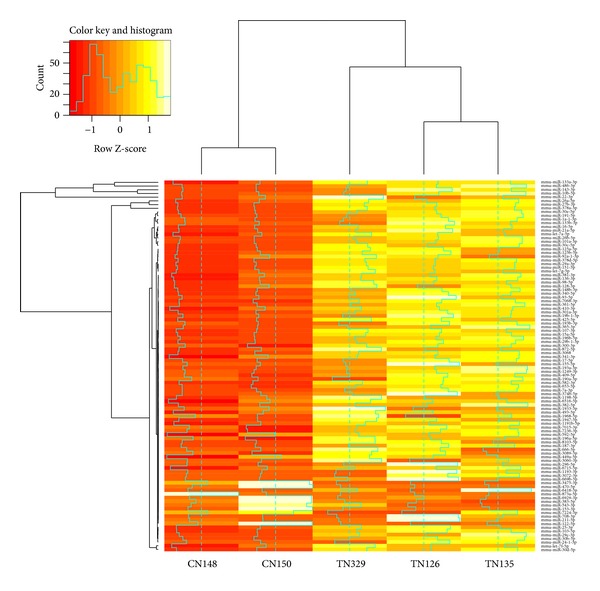
Identification of differentially expressed miRNAs in control and transgenic mice: Cluster analysis of differentially expressed miRNAs in control and transgenic mice based on the read counts obtained from deep sequencing data. CN: Control, TN: Transgenic.

To identify miRNAs associated with skeletal muscle hypertrophy, we compared the miRNA transcriptoms of skeletal muscle between MSTN propeptide transgenic mice and their littermate control mice. Of the 461 known miRNAs, 57 differentially expressed miRNAs were identified (Additional File 4). The miRNAs, which satisfied the criteria log2FoldChange ≥ 1 or ≤−1 and *P* ≤ 0.05, *Q* value 0.1, were denoted as differentially expressed miRNAs. Cluster analysis was generated to check the expression of miRNAs in different samples (Figure 5).

**Figure 6:**
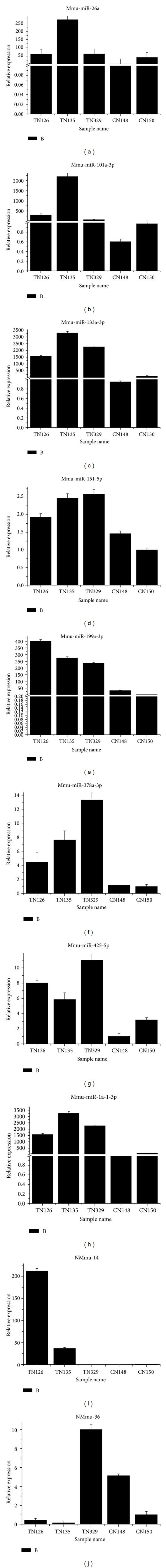
Validation of differentially expressed known and novel miRNAs: (a)–(j) eight differentially expressed and two novel miRNAs were selected for sequencing results validation.

To validate the differentially expressed miRNAs of the wild-type control and transgenic mice, 8 known miRNAs and 2 novel miRNAs were randomly selected and their expression levels quantified by using real-time RT-PCR (Figures 4 and 6). Of the 8 known miRNAs examined, 7 miRNAs (miR-425, miR-26a, miR-1a, miR-199a, miR-101, miR-378, and miR-151) showed a consistent pattern with the deep sequencing data (Figures 6(a)–6(j)). These data demonstrated that deep sequencing is a sensitive and reliable method for identifying differentially expressed miRNAs.

### 3.6. Target Prediction for Differentially Expressed miRNAs

To ascertain the query how miRNAs function in concert with their target genes in muscle tissue, we identified targets of the differentially expressed miRNAs. Of the 57 differentially expressed miRNAs, 20 abundant miRNAs were selected to predict the targets and 4,413 annotated mRNA transcripts were identified (Additional File 5).

### 3.7. Gene Ontology and Pathway Analysis 

**Figure 7:**
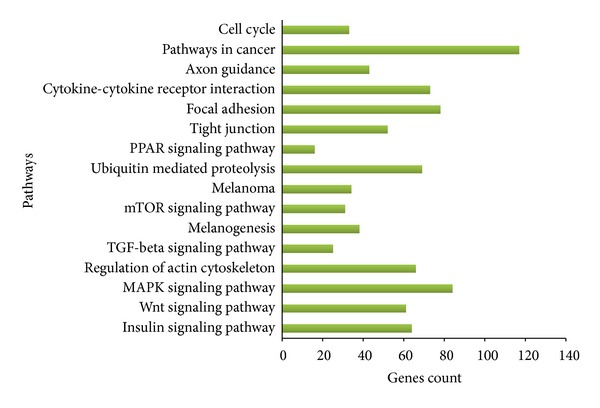
Pathways with respective gene numbers: top 16 Pathways predicted to be targeted differentially expressed miRNAs given on *Y*-axis and genes count on *X*-axis.

Predicted 4,413 targets were analyzed for pathway analysis and the results showed that approximately 110 pathways are involved in corresponding miRNAs. Out of 110 pathways, 16 pathways are found to be associated with the most number of targeted genes (Figure 7), including mTOR signaling, insulin-like growth factor, MAPK signaling, and TGF-*β* signaling. We also found that 20 differentially expressed miRNAs target many important genes such as FST, SMAD3, SMAD4, TGFBR1, ACRVR1a, ACVR1C, ACVR2a, ACVR, AKT3, and MEF2C (Table 4), which have been known to play a vital role in MSTN signalling pathway.

### 3.8. Results of Q-PCR Validation

Out of 57 differentially expressed miRNAs, 8 known miRNAs and 2 novel miRNAs were confirmed by using real-time RT-PCR (Figures 6(a)–6(j)). Out of 8 known miRNAs, 6 miRNAs have been functionally linked to myogenesis (i.e., miR-1a, miR-26a, miR-133a and miR-199a, miR-101, and miR-378 [38, 50, 51, 54, 55, 70]). The levels of these miRNA expressions from Q-PCR analysis are consistent with the RNA sequence data in both wild-type and transgenic mice.

## 4. Discussion

### 4.1. miRNA Identifications from Myostatin Propeptide Transgenic and Wild-Type Mice

Direct sequencing of RNA molecules with next-generation sequencing (NGS) technology has revolutionized the analysis of transcriptome due to high-throughput scale and low cost. Recent development with Illumina's microRNA-Seq by Genome Analyzer System had enabled direct identification and profiling of microRNAs in various organisms [71]. The present study identified a total of 461 miRNAs, of which 57 were known and differentially expressed and 69 were novel in skeletal muscle tissue. Of the 57 differentially expressed miRNAs mmu-mir-133a was the most abundant in the wild-type control (32, 12,465.607) and transgenic (57, 10,658.17) mice. It is previously reported that miR-133a enhances myoblast proliferation by repressing serum response factor (SRF) [23]. miR-206 was demonstrated to be the most abundant miRNA in skeletal muscles of broilers (131,609 reads) and layers (222,998 reads) [48]. Considering these previous studies, we presume that miR-133a and miR-206 play an important role in skeletal muscle development. In our study, we observed that miR-206 expression level was significantly lower than miR-133a in comparison to previous studies. miR-206 is reported to be one of the skeletal muscle-specific myomiR and many studies have documented its pivotal role in skeletal muscle differentiation [43, 49, 72–75] and miR-133a in regulating myogenesis by increasing muscle cell proliferation [23, 38, 76]. Differences in the expression levels of both the miRNAs (miR-133a and miR-206) in skeletal muscle libraries could reflect different roles of these miRNAs in terms of myogenesis regulation [77]. Similar to miR-133a and miR206, miR-1 also regulate muscle differentiation and development [23, 77–79]. miR-1 and miR-133 modulate skeletal-muscle-cell proliferation and differentiation by repressing the activity of HDAC4 (histone deacetylase 4; a signal-dependent inhibitor of muscle differentiation) and SRF, respectively, thereby establishing negative-feedback loops for muscle-cell differentiation [23].

In addition to well-known myomiRs, recent studies have demonstrated that miR-486 [49], miR-378 [50], miR181a [80], miR-21a, miR-101a, and miR-151 [54] are also involved in regulation of myogenesis and several other ubiquitously expressed miRNAs have also been found to participate in myogenesis, including miR-26a [51], miR-27b [52, 53], and miR-29 [44]. All these nine miRNAs were also found to be abundantly expressed in our sequence libraries, which indicated their important roles in the hypertrophic skeletal muscle of the myostatin propeptide transgenic mice.

### 4.2. Differentially Expressed miRNA Responsible for Skeletal Muscle Hypertrophy

A total of 57 differentially expressed miRNAs were identified; out of these miR-133a, miR-378a, and miR-26 were highly abundant. MiR-133a [23, 38], miR-378a [50], miR-26a [51], miR-27b [52, 53], miR-21a [56], miR-29a [44], miR-148 [58], and miR-103 are skeletal muscle specific miRNAs and play a vital role in muscle differentiation and proliferation as reported in previous studies. miR-181 is upregulated during myocyte differentiation and represses homeobox protein Hox-A11, a repressor of muscle-cell differentiation, thereby allowing new muscle growth [81]. The expression of miR-133 (miR-133a, miR-133b), miR-1, and miR-181 (miR-181a, miR-181b, and miR-181c) was profiled in muscle from patients affected by myotonic dystrophy type1 and it was observed that they were specifically induced during myogenesis [82]. miR-148a promotes myogenic differentiation and downregulates ROCK1 gene at the translational level and plays a positive role in skeletal muscle development through the RhoA/ROCK pathway [58].

**Figure 8:**
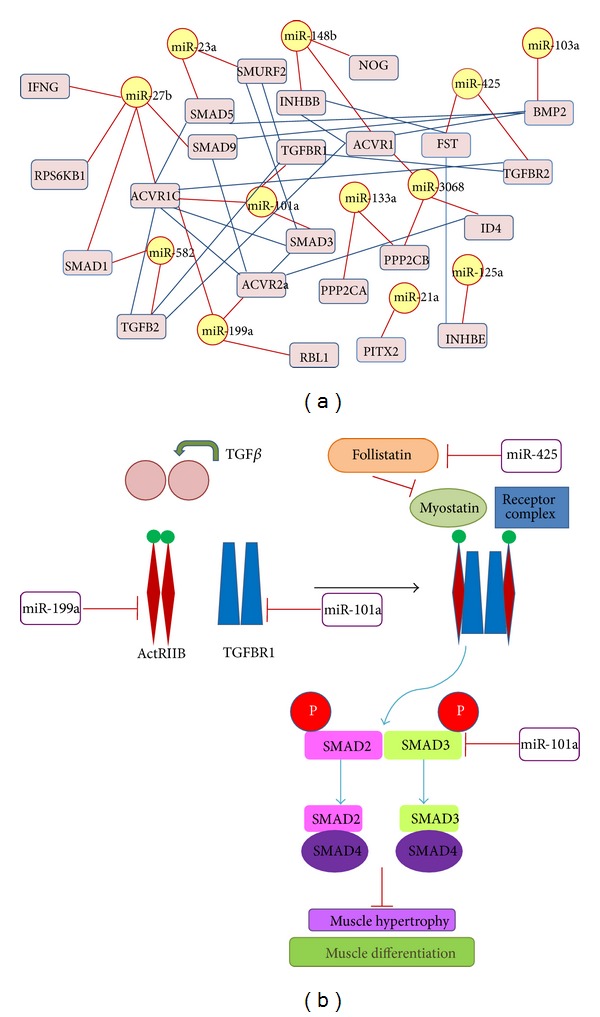
(a) Interaction network of differentially expressed miRNAs and their targets: network construction can be divided into two components: miRNA-target and target-target interaction, and candidate miRNA targets were predicted by mRDB. Target-target pairs interaction were searched from STRING database. In the network yellow nodes represented miRNAs and pink nodes represented targets, red lines denote miRNA-target interaction, and blue line denotes target-target interaction. These differentially expressed miRNAs predicted to be involved in TGFbeta signaling pathway. (b) MSTN signaling in skeletal muscle and miRNAs involved in the pathway: TGFb legend binds to the activin receptor two beta which interact with the TGFBR1 and they form a receptor complex, and mature myostatin inhibited by follistatin binds to the receptor complex which in turn phosphorylates regulatory SMADs, such as SMAD2 or 3. Phosphorylated regulatory SMADs are recognized by and associated with mediator co-SMADs, such as SMAD4, and translocated into the nucleus to interact with target gene promoter* cis*-elements and to regulate myostatin-specific gene expression. miRNAs were predicted to regulate the genes in TGFb signaling.

MiR-1 and miR-133a were proposed to contribute in muscle hypertrophy by the removal of their transcriptional inhibitory effect on growth factors such as IGF-1. Likewise, a regulatory feedback loop was demonstrated* in vitro *where IGF-1 downregulated miR-1 via the Akt/FoxO3a pathway [55]. It was shown that FoxO3a increased the levels of miR-1 resulting in reduced IGF-1 protein levels. The results from this study suggest that a number of differentially expressed miRNAs that could exert novel functions in skeletal muscle development. We identified targets for 20 highly abundant differentially expressed miRNAs and predicted genes were analyzed to search the pathways. Sixteen pathways were selected including mTOR signaling, insulin-like growth factor, MAPK signaling and TGF-*β* signaling and other pathways such as ubiquitin mediated proteolysis, focal adhesion, Wnt signaling pathway, regulation of actin cytoskeleton, cytokine-cytokine receptor interaction, tight junction, and cell cycle which are known to be involved in cell and tissue structure. Of these, we particularly focused on TGF-*β* signaling, as many studies reported that TGF-*β* family plays important roles in regulating muscle growth and development [83, 84]. Pathways analysis predicted that miR-101a is targeting TGFBR1 and SMAD3, miR-582 is targeting TGFB2, SMAD1, miR-425 is targeting TGFBR2 and FST, miR-199a is targeting ACVR2a, miR-148 is targeting ACVR1, and miR-103 is targeting BMP2 in TGF-*β* signaling pathway. A network was constructed to show the miRNA-target and target-target interaction in TGF-*β* signaling (Figure 8(a)). Myostatin (MSTN) is predominantly expressed in skeletal muscle and plays a crucial role in muscle development and also signals through the TGF-*β* branch [85] (Figures 8(a) and 8(b)). Myostatin binds to the TGFBR1 and makes a receptor complex for binding with ActRIIB, and activation of this receptor complex leads to the phosphorylation of SMAD2 and SMAD3.

## 5. Conclusion 

This study was carried out to identify differentially expressed miRNA transcriptome in myostatin propeptide transgenic mice and to evaluate the miRNAs associated with hypertrophic skeletal muscle. Predicted target and pathway analysis concluded that miR-101a is targeting TGFBR1 and SMAD3, miR-425 is targeting TGFBR2 and FST, miR-582 is targeting SMAD1 and TGFB2, miR-148 is targeting ACVR1, and miR-199a is targeting AcvR1a gene in the TGF-*β* signaling pathway. As far as we have known, these miRNAs and their gene targets, which are crucial to myostatin signaling pathway, have not been reported earlier. Annotations of these major miRNAs to the myostatin signal pathway in relation to skeletal muscle hypertrophy can provide important guidance for the application of miRNA approaches to enhancing skeletal muscle growth and development.

## Supplementary Material

Additional File 1: A Total of 461 mature know miRNAs were identified, their consensus sequence and homology miRNA in miRBase are mentioned in the list. These 461 miRNAs obtained in the sequencing data matched perfectly with 1908 known Mus musculus miRNAs in the miRBase. Mature read count and expression level of each miRNA is listed here in the file. Log2 fold change and P value was calculated for each identified known miRNA.
Additional File 2: List of Identified IsomiRs of different miRNAs which were having heterogeneity at 5' and 3' end from their miRBase reference sequences. IsomiRs were selected which were having most abundant isoform or more than one abundant isoform. Six isomiRs i.e. mmu-miR-16a-2, mmu-miR-133a-1, mmu-miR-188b-2, mmu-miR-199a-2, mmu-miR-486 and mmu-miR-26b-5P were selected.
Additional File 3: List of predicted Novel miRNAs. A total of 69 Novel miRNAs were identified out of which some were having high read count in control and in transgenic library. Two novel miRNAs i.e. NMmu-14 and NMmu-36 which were having more than 100 read counts in control and transgenic library were selected for validation.
Additional File 4: After analysing miRseq data using miRDeep2 software a total of 57 differentially expressed miRNAs were identified. On the basis of each miRNA read count and expression level log2fold change and P value was calculated. Differentially expressed miRNAs were identified with satisfied criteria log2FoldChange ≥ 1 or ≤ -1 and P ≤ 0.05, Q value 0 .1.
Additional File 5: Randomly 20 abundant miRNAs were selected and their targets were identified using mRDB online tool. A total of 4,413 targets were identified for 20 miRNAs. We found some of miRNAs targets, which are known to play important role in TGF-*β* signaling.

## Figures and Tables

**Table 1:** Number of miRNA reads from control and transgenic libraries.

Reads	CN148	CN150	TN126	TN135	TN329
Total reads	11303077	9357529	9734519	10207035	21774919
Clean reads	10479962	8375356	9052728	9677365	20070380
Qualified%	92.728%	89.504%	92.996%	94.812%	92.172%
Mapped reads	536489	224550	177471	190093	429933
Mapped%	5.119188	2.68108	1.960415	1.964305	2.142126856

**Table 2:** Highly abundant known miRNAs in control and transgenic libraries.

miRNA	Reads					
	CN148	CN150	TN126	TN135	TN329	Previous studies in skeletal muscle
mmu-miR-22-3p	1637847	924589	1186390	755381	1123582	
mmu-miR-133a-3p	1143874	716877	984332	1004715	1049011	[38]
mmu-miR-486-3p	694107	288434	927009	902799	1325315	[49]
mmu-miR-143-3p	496985	195585	820161	849193	676631	
mmu-miR-378a-3p	427775	201482	420654	356711	417747	[50]
mmu-miR-10b-5p	326382	192684	699540	645680	909894	
mmu-miR-26a-5p	265020	151950	297146	184254	263216	[51]
mmu-miR-27b-3p	190947	85527	204496	151307	174403	[52, 53]
mmu-miR-10a-5p	106751	35960	218660	179584	198961	
mmu-miR-126a-5p	104630	34633	173507	98023	148161	
mmu-let-7f-5p	76751	57966	85569	62527	66748	
mmu-miR-30d-5p	71324	36990	71091	53116	69360	
mmu-miR-30e-5p	46295	22850	77046	49869	64934	
mmu-miR-191-5p	43014	20310	61773	53825	66552	
mmu-miR-30c-5p	37487	18170	36383	25658	36686	
mmu-miR-16-5p	28759	13306	33389	30684	29020	
mmu-miR-101a-3p	27883	12254	42753	32687	43902	[54]
mmu-let-7i-5p	27804	25012	21811	16454	15724	
mmu-miR-100-5p	26629	8764	62824	58392	50326	
mmu-miR-1a-1-3p	25824	4872	60020	59300	56369	[55]
mmu-miR-21a-5p	25762	13328	32285	31527	22657	[56]
mmu-let-7a-5p	22645	14527	26374	19001	18235	
mmu-miR-125a-5p	22035	10736	20767	16909	13863	[57]
mmu-miR-127-3p	20888	19550	42560	28964	36344	
mmu-miR-125b-5p	18733	10574	17810	18525	12921	[57]
mmu-miR-26b-5p	18498	7630	27992	20838	26573	
mmu-miR-378d-5p	17270	7758	20914	14457	17438	
mmu-miR-133b-3p	17250	7097	41049	66977	46252	[38]
mmu-miR-92a-1-3p	16783	12552	11462	14209	10021	
mmu-miR-148a-3p	16589	9216	20184	35176	17861	[58]

**Table 3:** Abundantly expressed novel miRNAs in control and transgenic libraries.

Name	Mature sequence	Reads					Chromosome position	Strand
		CN148	CN150	TN126	TN135	TN329		
NMmu-14	ugauuggaagacacucugcaaca	0	0	159	129	0	16	—
NMmu-36	gauucggcugaucuggcuggc	457	0	0	0	515	14	—

**Table 4:** Abundant differentially expressed miRNAs their targets and predicted pathways for targets.

	miRNAs	MAPK signaling pathway	TGF-*β* signaling pathway	mTOR signaling Pathway
1	miR-27b	EGFR, RPS6KA5, KRAS, SOS1, MAP2K4, MKNK2, CACNG2, FGF1, RAPGEF2, CACNA1A, ACVR1C, PRKCB	SMAD9, IFNG, RPS6KB1, SMAD1, ACVR1C	PDPK1, RPS6KB1, RICTOR
2	miR-16a	CACNA2D1, FGF9, NF1, GNA12, RAF1, MKNK1, MAPK8, AKT3		PIK3R1, AKT3
3	miR-21a	MAP3K7, NTF3, FASL, MAPK10, DUSP8	PITX2	
4	miR-23a	PTPN7, MAP4K4, MAP3K5, PAK2, RRAS2, CACNA1E, FAS, STK4, CHUK	SMAD5, SMURF2	PIK3CB, PIK3R3
5	miR-26a	RPS6KA6, RAP1A		RPS6KA6, ULK1, ULK2
6	miR-29b	DUSP2, GNG12		VEGFA, IGF1, EIF4E2
7	miR-30b	TAOK1, RASA1, MAP3K12		PIK3CD, PRKAA2
8	miR-30c	TAOK1, RASA1, MAP3K12		PIK3CD, PRKAA2
9	miR-30d	TAOK1, RASA1, MAP3K12		PIK3CD, PRKAA2
10	miR-30e	TAOK1, RASA1, MAP3K12		PIK3CD, PRKAA2
11	miR-101a	DUSP1, NLK, TGFBR1, CACNB2, FGF10	TGFBR1, SMAD3	HIF1A, PRKAA1
12	miR-103	PRKCA, CDC25B	BMP2	CAB39L, PGF
13	miR-125a	TRAF6, MAP3K11	INHBE	
14	miR-133a	ARRB1, B230120H23RIK, FGF12, MECOM	PPP2CA, PPP2CB	
15	miR-148b	SOS2, IKBKB, GADD45A	INHBB, NOG, ACVR1	ULK3, RICTOR, MLST8
16	miR-199	MAP3K4, ACVR1C	ACVR2A, RBL1, ACVR1C	MTOR
17	miR-378a	CACNG8, RASGRF1, ELK4, PLA2G12A, TRAF6		
18	miR-425a	MEF2C, DUSP2, MAP2K1, TGFBR2, IL1A	TGFBR2, FST	IGF1, CAB39, FIGF
19	miR-582	BDNF, PPM1B, RASA2, TGFB2	SMAD1, TGFB2	
20	miR-3068	RASGRP3, MAPT, FGF12, CACNA1C, IL1A	PPP2CB, ID4, ACVR1	PIK3CA
